# Extracellular Vesicles-Based Cell-Cell Communication in Melanoma: New Perspectives in Diagnostics and Therapy

**DOI:** 10.3390/ijms24020965

**Published:** 2023-01-04

**Authors:** Katarzyna Kluszczynska, Malgorzata Czyz

**Affiliations:** Department of Molecular Biology of Cancer, Medical University of Lodz, 6/8 Mazowiecka Street, 92-215 Lodz, Poland

**Keywords:** biomarker, cell-cell communication, drug resistance, exosomes, extracellular vesicles, melanoma, miRNA

## Abstract

Extracellular vesicles (EVs) are a heterogeneous group of cell-secreted particles that carry cargo of functional biomolecules crucial for cell-to-cell communication with both physiological and pathophysiological consequences. In this review, we focus on evidence demonstrating that the EV-mediated crosstalk between melanoma cells within tumor, between melanoma cells and immune and stromal cells, promotes immune evasion and influences all steps of melanoma development from local progression, pre-metastatic niche formation, to metastatic colonization of distant organs. We also discuss the role of EVs in the development of resistance to immunotherapy and therapy with BRAF^V600^/MEK inhibitors, and shortly summarize the recent advances on the potential applications of EVs in melanoma diagnostics and therapy.

## 1. Introduction

**Figure 1:**
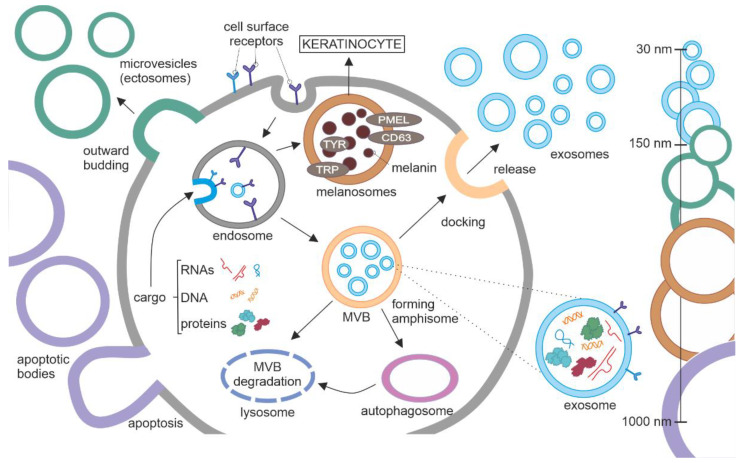
Biogenesis of extracellular vesicles (EVs). EVs can be categorized based on their size as follows: (1) apoptotic bodies, the largest (1–4 μm in diameter); (2) ectosomes/microvesicles belong to the group medium/large EVs (0.1–1 μm), that are shed directly by the plasma membrane, and their specific cargo accumulate at the cytosolic surface; (3) exosomes are small EVs (30–150 nM) that are released by the multivesicular body (MVB) fused with the plasma membrane. Several steps can be distinguished in exosome biogenesis. Proteins, lipids, nucleic acids, and metabolites (cargo) from the cytosol accumulate in the early endosome by inward budding. MVBs can be proceeded in alternative directions, (1) towards the plasma membrane to release exosomes to extracellular fluids; (2) towards lysosomes for degradation; (3) towards autophagosome to form amphisome for secretory autophagy or lysosomal degradation. Melanosomes are produced by melanocytes and transferred to keratinocytes to protect the skin from UV radiation. Similarly, to exosomes, melanosomes are derived from the endosomal membrane. They are an example of cell-type specific EVs, in terms of structure and function. Abbreviations: CD63, a member of the tetraspanin superfamily of activation-linked cell surface antigens; PMEL, pre-melanosome protein; TRP, tyrosinase-related protein; TYR, tyrosinase.

According to the guidelines of the International Society of Extracellular Vesicles (ISEV) [1], extracellular vesicles (EVs) are defined as particles naturally released from the cell that are delimited by a lipid bilayer and cannot replicate, i.e., do not contain a functional nucleus [1]. EVs can be generated by almost all cell types and identified in various biofluids such as saliva, plasma, serum, urine, breast milk, amniotic fluid, ascites, and cerebrospinal fluid. EVs can be classified based on their size, density (low/high), biogenesis, method of release, biochemical composition, tissue and cellular origin, and function [1]. Considering the first criterium, EVs are identified as exosomes (30–150 nmin diameter), microvesicles (0.1–1 μm), and apoptotic bodies (1–4 μm) [2]. While microvesicles and apoptotic bodies are shaped by direct budding of the plasma membrane, exosomes are formed in multivesicular bodies (MVBs) that arise in the endosomal compartment (Figure 1). They are secreted by fusion of MVBs with the plasma membrane. Alternatively, MVBs can fuse with lysosomes for vesicles degradation and recycling of their cargo, or MVBs can fuse with autophagosome forming amphisomes that can either secrete their cargo to extracellular fluid or undergo degradation in lysosomes (reviewed in [3]). In practice, assigning EVs to a particular biogenesis pathway remains difficult because of experimental limitations [4]. Methods of EV isolation are based on easily distinguished features, such as size and density, but these features are not restricted to specific subtypes of EVs. A mixture of exosomes, HDL, small apoptotic vesicles, and vesicles blebbing from cell membrane can be found in the same range of size. Therefore, it is difficult to isolate exosomes from a whole subpopulation of similarly sized vesicles and provide a reliable characterization of exosomes across the range of co-isolated vesicles. EVs’ biogenesis and composition of their cargo have been reviewed elsewhere [5,6,7,8]. In this review, the term “exosomes” is used only when vesicles were identified as exosomes (recently named small extracellular vesicles), and the term “extracellular vesicles” is referred to vesicles without a defined origin. EVs mediate intercellular communication affecting various physiological and pathophysiological processes. They carry diverse molecular cargoes, including proteins, nucleic acids, lipids, and metabolites, both common and unique for cells of origin, their phenotypes, metabolic state, and function. By transferring their cargo, EVs regulate recipient cell function. They play important roles in angiogenesis [9,10], the regulation of metabolism [11], fertilization [12], trophoblast implantation [13], pregnancy [14], neuronal differentiation [15], regeneration, synaptic plasticity, neural trophic support, the regulation of myelination [16], dendritic spine formation, maintaining blood-brain barrier integrity [17], and immunomodulation, including the suppression of the maternal immune system by the fetus and the modulation of the infant’s immune system by maternal milk-derived exosomes [18,19,20]. The involvement of intercellular communication through exosomes in pathological processes, particularly carcinogenesis, but also neurodegeneration and inflammatory diseases, has attracted great attention of researchers. EVs might provide several useful information on cell status, however, their potential roles are still underestimated, due to the low level of specificity of the obtained samples.

Several EVs are cell-type specific. Melanosomes are a good example, as they are exclusively produced by melanocytes and transferred to keratinocytes (Figure 1) [21,22]. Similarly, as exosomes, they are derived from endosomal membrane [23]. They contain several specific markers such as tetraspanin CD63, premelanosomal protein (PMEL), tyrosinase, tyrosinase-related protein 1 and 2 (TRP1 and TRP2, respectively), some of them participating in the synthesis of melanin, a pigment that protects skin from UV radiation.

In this review, we focus on the role of EVs in melanoma. A melanocyte-derived melanoma is the most lethal of cutaneous cancers, and in the majority of cases is associated with the mutation-deregulated activity of the RAS-BRAF-MEK-ERK pathway. While the incidence of melanoma continues to increase, the mortality from the metastatic disease remains unaltered [24]. This is in part due to advances in treatment, including immunotherapies with checkpoint inhibitors and targeted therapies against mutated B-Raf proto-oncogene (BRAF^V600^) and mitogen-activated protein kinase kinase 1/2 (MEK1/2) [25]. Despite the development of various therapies, the majority of melanoma patients still require additional treatment due to intrinsic or acquired resistance to therapy [26]. Therefore, in addition to reviewing the role of EVs in metastatic melanoma, we discuss their contribution to the development of resistance to currently used immunotherapy and targeted therapy with BRAF^V600^/MEK1/2 inhibitors. Our review also shortly summarizes recent advances in applying EVs as a source of potential biomarkers and delivery vehicles for novel therapeutics against melanoma.

## 2. Role of EVs in Human Skin Homeostasis

The human skin is made up of three layers, the epidermis, dermis, and hypodermis (fatty layer). Melanocytes surrounded by keratinocytes form the epidermal-melanin units in the epidermis, whereas fibroblasts can be found within the dermis layer. To maintain skin homeostasis, keratinocytes, melanocytes, and fibroblasts constantly communicate through direct contact, exosomes, and secreted factors. Keratinocytes modulate various activities of melanocytes, including melanin synthesis and its transfer from melanocytes to keratinocytes in melanosomes, and exosomes released by keratinocytes participate in the regulation of these processes [27,28]. Keratinocyte-derived exosomes can also modulate the function of dermal fibroblasts [29,30], and EVs derived from dermal fibroblast can affect keratinocytes [31,32].

While both UVA and UVB irradiation trigger skin pigmentation as the result of crosstalk between melanocytes and keratinocytes within the epidermal-melanin unit, it has been demonstrated that UVA and UVB irradiation can also induce different cellular responses [21,33]. UVA, but not UVB, causes the shedding of EVs from the plasma membrane of melanocytes that are endocytosed by keratinocytes enhancing antiapoptotic signaling in these cells [33]. The plasma membrane damage associated with EV-shedding is repaired by Ca2+-dependent lysosomal exocytosis [21].

## 3. Role of EVs in Melanoma Progression

**Figure 2:**
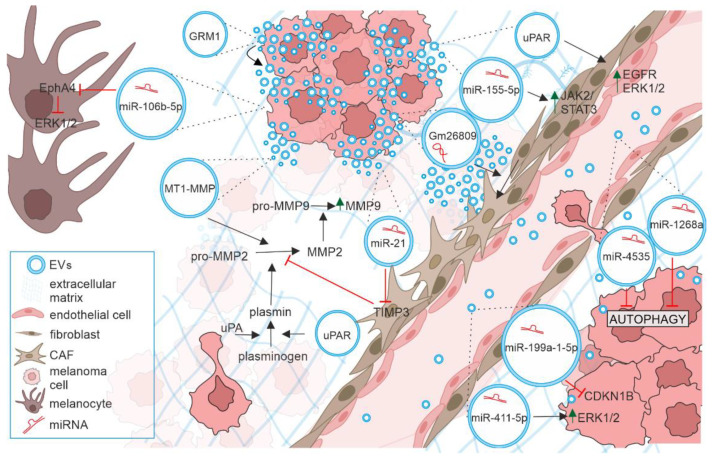
The role of extracellular vesicles in progression of melanoma. EVs (blue circles) released by melanoma cells can transfer their cargo such as miRNAs and proteins to various recipient cells. By modifying functions of melanocytes, other melanoma cells, stromal cells, and immune cells they contribute to all steps of metastasis including extracellular matrix remodeling, angiogenesis, pre-metastatic niche formation and proliferative colonization of distant organs. See text for details.

The development of cancer is a multistep process, in which cell-cell communication plays a pivotal role. EVs markedly contribute to all steps of the tumor development, from setting up a pre-cancerous environment to metastatic spreading by transferring information among cancer cells and between cancer cells and neighboring non-tumor cells, such as fibroblasts, endothelial cells, and immune cells or cells in the distant organs (reviewed in [34,35,36,37,38]) (Figure 2). Therefore, melanoma exosomes have been called ‘messengers of metastasis’ [39]. Two different modes of transfer can be distinguished: (1) by the internalization of EVs by the recipient cells that is called horizontal transfer; or (2) by the ligand-receptor interaction at the cell surface [40,41]. An interesting issue is how pro-invasive exosomes arise. It was demonstrated that a subset of melanomas overexpressed the GTPase RAB27A, thus generating pro-invasive exosomes, which correlated with poor survival of melanoma patients [42].

CDKN1B, Cyclin-Dependent Kinase Inhibitor 1B; EGFR, Epidermal Growth Factor Receptor; EphA4, EPH Receptor A4; ERK1/2, Extracellular Signal-Regulated Kinases 1/2; EVs, Extracellular Vesicles; GRM1, Glutamate Metabotropic Receptor 1; JAK/STAT, Janus Kinase/Signal Transducer and Activator of Transcription; MAPK, Mitogen-Activated Protein Kinase; MMP-2, and MMP-9, Matrix Metalloproteinases 2 and 9; MT1-MMP, Membrane Type-1 Matrix Metalloproteinase; TIMP3, Tissue Inhibitor of Metalloproteinase 3; uPA, urokinase Plasminogen Activator; uPAR, urokinase Plasminogen Activator Receptor; VEGF, Vascular Endothelial Growth Factor A.

### 3.1. EV Cargo Transfer from Melanoma Cells to Melanocytes

It was demonstrated in an early study that normal melanocytes could become invasive when exposed to exosomes from melanoma cells [43]. Exosomes derived from melanoma cells could communicate with the nearby melanocyte to promote epithelial-mesenchymal transition in primary melanocytes, which was regulated by let-7i and the MAPK pathway [44]. Recently, exosomal miR-106b-5p derived from melanoma cells was shown to activate ERK pathway in melanocytes by targeting Eph receptor A4 (EphA4), which resulted in the downregulation of E-cadherin level and upregulation of mesenchymal proteins (N-cadherin and fibronectin), thus promoting an invasive capacity of melanocytes [45].

### 3.2. EV Communication between Melanoma Cells

The transfer of ‘metastatic message’ via exosomes between melanoma cells was detected in several experimental settings. Horizontal transfer of proteins such as glutamate metabotropic receptor 1 (GRM1) carried by EVs from GRM1-positive cells promoted migration, invasion, and anchorage-independent growth of melanoma GRM1-negative cells [46]. miR-21 was reported to downregulate the level of tissue inhibitor of metalloproteinase 3 (TIMP3) and upregulate the expression of matrix metalloproteinases (MMPs) involved in ECM degradation [47]. The elevated levels of miR-4535 [48] and miR-1268a were found in highly metastatic melanoma cells [49], and these exosome-encapsulated miRNAs were shown to augment the colonization capability by inactivating the autophagy pathway in low metastatic melanoma cell line. The exosomal transfer of miR-411-5p from high- to low-metastatic melanoma cells enhanced the proliferative colonization capacity in the latter by activation of the ERK pathway [50]. Exosomal crosstalk between melanoma cells with high and low metastatic potential promoting invasiveness was demonstrated as delivery of functional miR-199a-1-5p, which participated in the inactivation of cyclin-dependent kinase inhibitor 1B (CDKN1B), a cell cycle inhibitor [51]. CDKN1B was shown to be downregulated and the PI3K/AKT pathway activated in primary melanoma cells after exosomal transfer of miR-222, which was accompanied by increased proliferation and induced invasive and chemotactic capabilities in primary melanoma cells [52].

### 3.3. Melanoma-Derived Exosomes in Promoting Angiogenesis, Pre-Metastatic Niche Formation and Proliferative Colonization by Affecting Extracellular Matrix Remodeling and Stromal Cell Functionalities

Extracellular matrix (ECM) is crucial for cancer cell dissemination. A melanoma-specific mechanism of EV release was shown to contribute to invasive potential of melanoma cells. Membrane-type 1 matrix metalloproteinase (MT1-MMP) participating in matrix degradation was found in melanoma EVs utilizing melanosome secretion pathway [53]. Several reports have demonstrated that melanoma-derived EVs can modify endothelial cells. Urokinase-type plasminogen activator receptor (uPAR)-positive EVs released by melanoma cells were involved in angiogenesis by overexpression of VE-Cadherin, EGFR, and uPAR and activation of ERK1/2 signaling in endothelial cells [54]. WNT5A expression when enhanced in metastatic melanoma was shown to induce the release of exosomes containing pro-angiogenic factors such as VEGF, IL-6, and MMP2, accompanied by endothelial cell branching [55].

Melanoma-derived EVs are involved in the generation of cancer-associated fibroblasts (CAFs) either from normal fibroblasts or from endothelial cells. The first mechanism can be exemplified by exosomal delivery of lncRNA Gm26809 to normal fibroblasts [56]. The second one has been demonstrated as melanoma-derived exosomes enhancing transdifferentiation of human umbilical vein endothelial cells (HUVECs) into CAFs [57,58]. It was shown that melanoma EVs stimulated the pro-inflammatory activity of CAFs [59]. The activity of fibroblasts could be promoted by melanoma EVs containing miR-21 [47] and miR-155-5p [60]. miR-155-5p was reported to induce the proangiogenic switch by activating the JAK2-STAT3 signaling pathway [60]. Bi-directional EV-mediated crosstalk between melanoma cells and CAFs was recently reviewed [61]. Melanoma-derived exosomes containing miR-155 and miR-210 were shown to reprogram dermal fibroblasts by increasing aerobic glycolysis and reducing oxidative phosphorylation, which led to extracellular acidification [62]. The role played by melanoma-derived EVs under acidic and hypoxic conditions has been recently reviewed [63].

The first evidence indicating that exosomes from melanoma cells can contribute to pre-metastatic niche formation by regulating the function of stromal cells at the distant organs, came from the study showing that melanoma-derived exosomes enhanced the migration of melanoma cells to these sites in sentinel lymph nodes that were enriched in melanoma exosomes [64]. Peinado et al. showed that exosome could “educate” bone marrow (BM)-derived dendritic cells (BMDCs) toward a pre-metastatic and pro-vasculogenic phenotype through the receptor tyrosine kinase MET [65]. In vivo study demonstrated that pre-education of BMDCs with exosomes from a highly metastatic melanoma was sufficient to accelerate tumor growth. BMDC-educated mice had increased size and number of metastases, enhanced recruitment of BMDCs, and higher tumor vascular density [65]. It was shown that lymph node metastatic melanoma cells expressed prominin-1 (CD133), and exosomes derived from these cells were enriched in this protein [66]. A transfer of prominin-1 from melanoma cells to bone marrow-derived stromal cells via exosomes was detected [66]. Recently, it was demonstrated that EVs from metastatic melanoma cell lines contained nerve growth factor receptor (NGFR), which was delivered to lymphatic endothelial cells and reinforced premetastatic niche formation in lymph nodes of murine model [67]. These EVs induced the activation of ERK and nuclear factor κB (NF-κB), and expression of intracellular adhesion molecule (ICAM-1) in lymphatic endothelial cells, thus enhancing lymphangiogenesis and melanoma cell adhesion [67]. In another study, EVs from melanoma cells transported by lymphatic vessels induced lymph node remodeling by selectively interacting with CD169+ macrophages and lymphatic endothelial cells, and the latter interaction was partly dependent on lymphatic expression of vascular cell adhesion molecule 1 (VCAM-1) [68]. EV-transferred melanoma antigens presented by lymphatic endothelial cells led to apoptosis of tumor specific CD8+ T cells and immune inhibition [68].

## 4. Melanoma-Derived EVs Modulate the Function and Antitumor Activities of Immune Cells

Growing evidence indicates that cancer-derived EVs possess immune-regulatory potential, which is a part of cancer survival strategy [69]. Cancer-derived EVs can modulate both innate and adaptive immune responses by delivering tolerogenic signals to immune cells [70]. EVs carry multiple biologically-active immunosuppressive molecules, such as CD39, CD73, Fas ligand (FasL), TGFβ, TNF-Related Apoptosis-Inducing Ligand (TRAIL), programmed cell death ligand 1 (PD-L1), and cytotoxic T-lymphocyte antigen 4 (CTLA-4), with the potential to simultaneously modulate several molecular pathways in recipient immune cells [71].

Melanoma-derived EVs can suppress the proliferation, function, and viability of CD8+ T cells along diverse mechanisms. EVs were shown to transfer Src homology 2 (SH2)-containing protein tyrosine phosphatase 2 (SHP2), both transcript and protein, from B16F0 melanoma cells to primary CD8+ T cells, thus inhibiting T cell viability [72]. Exosomes from the plasma of melanoma patients suppressed proliferation of CD8+ T cells and promoted their apoptosis by elevating the levels of TRAIL and FasL [71]. Melanoma-derived exosomes that carried PD-L1 on their surface also suppressed the function of CD8+ T cells [73]. Vignard et al. showed that miR3187-3p and miR-498 transferred from melanoma cells through exosomes to CD8+ T cells downregulated CD8+ T cell response through decreased T-cell receptor (TCR) signaling [74]. Exosomes isolated from serum of melanoma patients carried ectonucleotidase CD73 that contributed to the suppression of T cell function [75]. Most recently, it was demonstrated that melanoma-derived exosomes with intercellular adhesion molecule 1 (ICAM-1) on their surface could interact with lymphocyte function-associated antigen 1 (LFA-1) upregulated in activated CD8+ T cells, which was required prior exosomal PD-L1-mediated immune suppression [76]. The upregulation of LFA-1 and ICAM-1 promoted the interaction between T cells and exosomes, whereas the inhibition of ICAM-1 on melanoma-derived exosomes reduced this interaction and diminished PD-L1-mediated suppression of T cells [76]. ICAM-1 was earlier found important for internalization of exosomes by immature dendritic cells for antigen presentation to CD4+ T cells [77], whereas LFA-1 on activated CD4+ T cells was crucial for exosome binding [78]. ICAM-1 on exosomes derived from dendritic cells was required for efficient priming of naïve T cells [79]. CD4+ T cells were shown crucial for mediating anti-melanoma effects, and their depletion resulted in substantial diminution of antitumor effects of the vaccination with HCA587 protein [80].

Melanoma-derived EVs can affect other immune cells, such as macrophages and natural killer (NK) cells. T cell immunoglobulin and mucin-domain containing-3 (TIM-3) found in melanoma-derived exosomes suppressed the immune function of CD4+ T cells and induced the M2 polarization of macrophages [81]. Exosomes released by melanoma cells were shown to contribute to the induction of a tumor-promoting phenotype of Tumor Associated Macrophages (TAM) via exosomal miR-125b-5p targeting the lysosomal acid lipase A (LIPA) [82]. However, another report demonstrated that melanoma-derived exosomes could polarize macrophages not only in the M2 direction but also towards M1 phenotype exerting antitumor function [83]. Melanoma-derived EVs were found to diminish normal differentiation of circulating monocytes into dendritic cells, and instead a CD14+ immature myeloid population was generated with a TGF-β-mediated suppressive activity on proliferation and function of T cells [84]. TGF-β in melanoma-derived exosomes also contributed to the promotion of a suppressive phenotype of antigen-presenting cells (APCs) [85]. Exosomes isolated from the plasma of melanoma patients downregulated the expression of natural killer group 2, member D (NKG2D) in NK cells, thus reducing their antitumor activity [71].

## 5. EV Cargoes Contribute to Melanoma Resistance to Target Therapy

Several treatment options can be offered to patients with melanoma depending on the stage of disease, genetic background, and the place of malignant occurrence. Surgery is the first-choice treatment in the early stages of melanoma. In more advanced diseases, and if the malignant changes cannot be removed by surgery, targeted therapy or immunotherapy are mostly used (reviewed in [25,86]). FDA-approved targeted therapeutics against BRAF^V600^ (vemurafenib, dabrafenib, and encorafenib) and MEK1/2 (trametinib, cobimetinib, and binimetinib) can cause a rapid initial regression of metastatic BRAF^V600^ melanomas but acquired resistance to treatment is still a fundamental clinical challenge [26,87].

The resistance of melanoma to targeted therapies can be driven by genetic changes [88] and adaptive epigenetic alterations supported by the plasticity of cellular signaling and tumor microenvironment [89,90,91,92,93]. The role of tumor microenvironment in mediating epigenetic escape of melanoma cells from the BRAF^V600^/MEK inhibition is well proven by numerous studies as exemplified by [94,95,96,97,98,99,100]. The exosomal transfer of functional cargo is a part of the microenvironmental contribution to epigenetic changes crucial for the resistance to treatments. There are several levels of epigenetic regulation that can be modified in cancer, and non-coding RNAs, especially miRNAs are important epigenetic regulators markedly contributing to aberrant gene expression. miRNAs encapsulated within EVs were demonstrated in multiple studies to play a substantial role in cancer recurrence related to resistance to diverse treatment modalities (reviewed in [101]). Reports on the role of EVs-derived miRNAs in the development of resistance to targeted therapy in melanoma are summarized in Table 1.

It was shown that BRAF^V600^ inhibition with vemurafenib substantially increased the secretion of EVs from melanoma cells and significantly changed the RNA profiles in melanoma cells and melanoma-derived EVs [102]. EVs from vemurafenib-treated melanoma cells contained unique miRNAs, with much higher level of miR-211-5p. It was shown that the sensitivity of melanoma cells to this BRAF^V600^ inhibitor was reduced by transfection of miR-211-5p, whereas inhibition of miR-211-5p in vemurafenib-resistant cell line reduced their proliferation [102]. Svedman et al. showed that melanoma patients that had elevated levels of let-7g-5p and miR-497-5p in the plasma EVs, belonged to a group with a higher probability of response to inhibitors of the MAPK pathway and significantly longer progression-free survival [103]. miR-3613-3p is an example of miRs downregulated in exosomes derived from vemurafenib-resistant melanoma when compared to exosomes from melanoma cells prior to treatment [104]. Cell division cycle 7 (CDC7) was suggested as a potential target for this miR. An association was found between a low level of miR-3613-3p and hyperactivation of CDC7 and ERK1/2 in melanoma cells. The restoration of miR-3613-3p in resistant cells re-sensitized them to vemurafenib, which was accompanied by the inhibition of CDC7 expression and ERK1/2 activity [104]. The downregulation of miR-3613-3p was earlier reported in exosomes derived from resistant breast cancer cells [113].

The development of resistance to BRAF/MEK inhibitors was also monitored by the detection of BRAF splicing variants in the plasma of melanoma patients with objective response followed by disease progression [105]. The alternative splicing of BRAF is one of the mechanisms of acquired resistance to BRAF/MEK inhibition [114,115]. Circulating BRAF splicing variants were present in the plasma of three out of 38 patients with progressive melanoma, and these splicing variants were associated with EVs [105].

One of interesting questions related to the contribution of EVs to drug resistance is whether resistant phenotype can be transferred from drug-resistant cells to drug-sensitive cells within a tumor or to susceptible cancer cells at distant localization. The exosomal transfer of PDGFRβ is an example of how exosomes derived from melanoma cells resistant to a BRAF^V600^ inhibitor can support the development of resistance in drug-sensitive cells [106]. It was shown that the exosomal transfer of platelet-derived growth factor receptor beta (PDGFRβ) increased the viability of sensitive cells in the presence of the drug, which was associated with the activation of the phosphatidylinositol-3-OH kinase (PI3K)/serine/threonine kinase 1 (AKT) signaling. Moreover, treatment with PDGFRβ-neutralized antibodies abolished this effect [106]. The functional involvement of EVs in spreading of a drug resistant phenotype was suggested in the study showing an implication of a truncated form of anaplastic lymphoma kinase (ALK), named ALK^RES^, in resistance to BRAF^V600^ or MEK1/2 inhibitors [107]. EVs transporting active ALK^RES^ from vemurafenib-resistant melanoma cells to recipient cells sensitive to this drug were capable to re-activate the BRAF/MEK/ERK pathway in drug-treated sensitive cells [107].

Besides BRAF/MEK inhibitors, other drugs including alkylating agents are used in clinics in the treatment of melanoma patients. Temozolomide and cisplatin provided another example of how EVs shed by melanoma cells in response to treatment supported melanoma repopulation. It was demonstrated that EVs secreted by melanoma cells in response to temozolomide promoted a genetic reprogramming in melanoma cells in vivo by the upregulation of genes involved in cell survival, DNA repair, and proliferation [116]. Melanoma cells were not the only recipient cells as vesicles secreted after temozolomide treatment also induced the M2 pro-tumoral phenotype in macrophages [116].

## 6. Role of EVs in Resistance to Immune Checkpoints Inhibitors

Immunotherapy with immune checkpoint inhibitors (ICIs) largely contributes to increased survival rate of melanoma patients with metastatic disease, however, a subset of patients do not respond to this treatment [25]. Two classes of ICIs are approved by FDA to be used alone or in combination with other drugs: (1) antibodies against cytotoxic T-lymphocyte-associated protein 4 (anti-CTLA-4) that prevent CTLA-4 present on T cells from interacting with inhibitory signals on APCs; and (2) antibodies against programmed death protein 1 (PD-1) or its ligand PD-L1 that block the interaction of PD-1 receptor on T cells with ligands, PD-L1 and PD-L2 [117]. As several patients do not respond to immunotherapy, it would be helpful to find a predictor of therapeutic outcomes and acquisition of ICI resistance. Numerous studies have suggested that efficacy of ICIs may be affected by PD-L1- and PD-1-positive exosomes released from melanoma cells, also in response to ICIs (Table 1). PD-L1 antigen was detected on the surface of exosomes released by metastatic melanomas [73]. The efficacy of treatment with ICIs nivolumab and pembrolizumab could be associated with PD-L1 mRNA level in plasma-derived exosomes of melanoma patients [108]. As the PD-L1 mRNA level significantly decreased in melanoma patients responding to therapy and increased in patients that progressed during treatment, the authors suggest that PD-L1 mRNA level in plasma-derived exosomes could be used to monitor response of melanoma patients to the anti-PD-1 therapy although larger cohorts of melanoma patients will be needed to confirm this finding [108]. In a prospective study, Cordonnier et al. revealed that PD-L1-positive exosomes secreted by melanoma cells exerted immunosuppressive properties by inhibiting the activation of T cells [109]. Moreover, the exosomal PD-L1 was present in blood plasma despite their presence or absence in the tumor biopsy. Its level was significantly elevated during tumor progression, and decreased in tumor response to therapy, independently of the therapy used (anti-PD-L1 or targeted therapies). The changes in the level of PD-L1-positive exosomes were associated with overall survival and progression-free survival [109]. The most recent study aimed to correlate the amount of PD-L1-positive EVs, but also PD-1-positive EVs with the response of metastatic melanoma patients to ICIs [110]. It was shown that the circulating EVs could bind nivolumab, and therefore the level of PD-1-positive EVs could contribute to resistance to anti-PD-1 immunotherapy. In another approach evaluating the efficacy of anti-PD-1/PD-L1 treatment, the outcome of inhibition of the cystine/glutamate antiporter (xCT) was investigated in mouse model of ICI-treated metastatic melanoma [111]. Inhibition of xCT increased PD-L1 expression and its secretion via exosomes by melanoma cells, which led to M2 macrophage polarization and eventually induced anti-PD-1/PD-L1 treatment resistance [111]. Exosomal CD73 from serum of melanoma patients was associated with therapy resistance to pembrolizumab or nivolumab [75]. Urokinase-type plasminogen activator receptor (uPAR) expression on the surface of EVs was found in melanoma patients as another indicator of innate resistance to immunotherapy [112]. Patients that responded to treatment had a significantly lower level of uPAR-positive EVs in the plasma than non-responders.

## 7. EV-Derived Biomarkers of Melanoma Progression and Treatment Efficacy

As cancer is a systemic disease involving spreading cancer cells and cancer secretome to distant organs, biofluids containing circulating tumor cells (CTCs), circulating tumor DNA (ctDNA), and EVs, can serve as a source of information (biomarkers) confirming the presence and evolution of cancer, also in the response to treatment. Biomarkers identified in the circulating EVs of cancer patients at early stages of asymptomatic disease could be of especially high clinical value. EV-based blood biomarker classifier has been developed recently for early detection of pancreatic, ovarian, and bladder cancer [118]. While biofluids are valuable non-invasive liquid biopsies, there are several challenges of using biofluids as a source of cancer EVs, including the lack of efficient and cost-effective method of EVs’ isolation for clinical monitoring, the heterogeneity of EVs that are released from several types of cells, the contamination of samples with cells and other types of vesicles etc. [119,120]. Melanoma-derived EVs have been studied to identify biomarkers that indicate various stages of disease. The early detection of melanoma metastasis has been connected with the elevated level of MDA-9/syntenin and Glucose-Regulated Protein 78 (GRP78) in exosomes from serum samples [121]. Chondroitin Sulfate Proteoglycan 4 (CSPG4) [122] and Caveolin-1 (Cav-1) [123] have been proposed as potential EV biomarkers differentiating the EVs of melanoma patients from healthy donors. Moreover, exosomes expressing CaV-1 were significantly reduced in melanoma patients exposed to chemotherapy when compared with untreated patients [123]. Overexpressed in melanoma cells melanotransferrin (MTf, CD228) was efficiently sorted to exosomes [124], and plasma samples from melanoma patients displayed an enhanced level of exosomal MTf in comparison to plasma samples obtained from healthy donors [125]. The elevated level of a glycolytic metalloenzyme alpha-enolase (ENO1), a protein considered as a biomarker of many cancers [126,127,128,129] was observed in advanced stage melanoma cells in comparison to normal melanocytes [130]. The expression of ENO1 was markedly higher in EVs from uveal melanoma than in EVs from normal choroidal melanocytes [131]. Several studies assessing the level of PD-L1-positive and PD-1-positive EVs as potential biomarkers of melanoma patient response to ICIs have been already discussed (see Section 6). Most recently, the reduced level of pSTAT3 and elevated expression of PD-L1 have been detected in plasma-circulating EVs from melanoma patients with brain metastasis compared to patients without metastases to central nervous system [132]. It has been suggested that monitoring PD-L1 in the plasma could be a biomarker of response to therapy of melanoma patients with brain metastasis although the origin of EVs with PD-L1, but also pSTAT3 in circulation have not be identified [132].

Various types of RNAs and DNA fragments can be found in cargoes of EVs, and miRNAs are the most extensively studied [133,134,135,136]. Early studies revealed 228 miRNAs differentially expressed in EVs from melanocytes compared to EVs from melanoma cells [43]. Since then, several microarray analysis have been performed comparing, for example, miRNAs from EVs with miRNAs from donor melanoma cells [66], miRNAs from the EVs of melanoma cells grown either in normoxic or hypoxic conditions [137], and miRNAs from different subsets of EVs [138] (reviewed in [139]). The detection of miRNAs in EVs isolated from plasma or serum of melanoma patients is still very challenging. Using plasma-derived exosomes, it was shown that miR-17, miR-19a, miR-21, miR-126, and miR-149 were expressed at higher levels in the exosomes of patients with metastatic sporadic melanoma compared to exosomes from familial melanoma patients or control subjects [134]. Recently, based on the bioinformatic analysis of TCGA sequencing data, the average expression of miR-1914-3p was found higher in the plasma EVs from metastatic melanoma patients and high-risk patients, while miR-342-3p was downregulated in EVs from melanoma patients with worse prognosis when compared to healthy individuals [140].

It is thought that the molecular cargo of EVs can be also used to predict responses to targeted therapies. EV phenotyping has demonstrated a high potential for monitoring melanoma patient responses to targeted therapies [141]. The specific EV profiles associated with the development of drug resistance have been identified using an EV phenotype analyzer chip (EPAC), and this technology has been also used to differentiate between melanoma patients and healthy volunteers based on their plasma EV phenotypes [141].

While blood plasma and serum are the most used sources of EVs, it was shown recently that a biofluid collected after lymphadenectomy by lymphatic drainage was enriched in EVs compared with the plasma of those stage III melanoma patients [142]. Interestingly, isolated EVs exerted melanoma-specific protein signatures and BRAF^V600E^ mutation, which has been correlated with the risk of relapse after lymphadenectomy [142].

Analyses presented above, but not exclusively, have several limitations, including the total number of patients, heterogeneity of samples, the method used for isolation of elements of EV cargo, etc. Therefore, they can be rather treated as preliminary results requiring future in-depth research in order to identify biomarkers of different stages of melanoma.

## 8. EVs as Therapeutic Tools for Melanoma Treatment

EVs are notable candidates for therapeutic applications because they have a natural ability to carry biomolecules and they share several features with parental cells. The EVs of selected cell types are thought to be applied as therapeutic agents in immune therapy, vaccination trials, regenerative medicine, and drug delivery (for review [143,144,145,146,147,148,149]. For example, exosomes secreted by stem cells have wide regenerative potential, which could be utilized in several areas such as wound healing [150,151], muscle regeneration [152], or neuron regrowth after spinal cord injury [153]. Due to their biocompatibility and high stability in body fluids, EVs have attracted considerable interest as drug delivery vehicles. EVs can be loaded with diverse cargo such as hydrophobic compounds, for example porphyrins [154] and curcumin [155]), drugs such as paclitaxel [156], miRNAs [157], photosensitizer to phototherapy, including pyropheophorbide-a (PPa) [158]), and CRISPR-Cas9 components [159,160].

EVs can be modified to obtain better properties e.g., to increase their circulation time by modification with nanobody-PEG-lipids [161] or decorating them with ligands to apply “eat me/don’t eat me strategy” for drug delivery [162]. Fusion EVs with cargo-loaded liposomes allows the generation of hybrid EVs that benefit from both the biocompatibility of EVs and the easiness of loading [163]. The method of directly targeting the recipient cells is one of the most critical factors determining the application of EVs as a carrier of biomolecules. Several cells present specific surface proteins (e.g., integrins) that facilitate EVs to reach the target [164]. Integrins naturally direct EVs to cells displaying complementary molecules, ensuring the specificity of this mutual communication. The other crucial factor is the way of administration. The choice of administration method should be carefully considered depending on the goal to reach [165].

### 8.1. EVs as a Part of Antimelanoma Treatment in Preclinical Studies

The development of delivery systems of anticancer agents that could enhance the selectivity of targeting tumor cells is in the focus of studies using EVs as delivery vehicles. Tumor cells could capture their own EVs more efficiently than EVs derived from other cells [166]. Melanoma-derived EVs were efficiently uptaken by autologous melanoma cells to transfer their cargo [164]. Homing selectivity gives a plethora of opportunities to use autologous exosomes against melanoma. It was nicely proven by delivering melanoma-derived EVs, double-labelled with gold and fluorescent dyes to metastatic lung nodules [167]. Jiang et al. engineered melanoma exosomes by equipping them with tumor necrosis factor-related apoptosis-inducing ligand (TRAIL) and loaded them with triptolide (TPL) [168]. The intravenous injection of TRAIL-Exo/TPL significantly suppressed tumor progression and reduced the toxicity of TPL in nude mouse melanoma model [168]. Gu et al. also used triptolide as an anticancer agent but they loaded TPL into exosomes harboring a cRGD peptide, which targeted the αvβ3 integrin receptor overexpressed on melanoma cells. cRGD-Exo-TPL construct significantly inhibited melanoma growth in vivo and prolonged the half-life of TPL [169]. PEG-coated EVs were shown to deliver doxorubicin to B16-F10 murine melanoma models [170]. Encapsulating inorganic particles with defined functions in EVs opens new possibilities in therapies, as exemplified by gold nanoparticles/EVs hybrids administered to murine melanoma cells to focus and narrow exposure field used in phototherapy [171]. The effects of melanoma-derived exosomes as carriers of photosensitizers and delivery systems for photodynamic therapy was recently reviewed [172]

EVs with defined molecular cargo are thought to be used as therapeutic agents in immune therapy. In a murine model of melanoma, EVs harboring signal regulatory protein alpha (SIPRα) caused CD47 depletion increasing immune cell infiltration and CD8+ T cell-mediated immunity against non-treated tumors [173]. Morishita et al. showed that the intratumoral administration of genetically engineered melanoma cell-derived exosomes containing endogenous tumor antigens and immunostimulatory CpG DNA decreased melanoma growth and lung metastasis in mice [174]. EVs derived from genetically modified melanoma cells expressing the TNFSF ligands 4-1BBL and OX40L modulated the immune response, induced T cell proliferation, which induced antitumor cytotoxicity [175]. Kim et al. revealed that in the response to gamma radiation (ƴ-irradiation) stressed melanoma cells produced exosomes that stimulated DCs maturation and conferred tumor growth inhibition [176]. Horrevorst et al. designed a DC-targeting tumor vaccine through glycan modification of melanoma-derived ApoEVs, which facilitated an uptake of tumor-derived (neo-) antigens by monocyte-derived DCs and enabled tumor-specific CD8+ T cell priming [177]. Labani-Motlag et al. used EVs as a platform potentially useful for virotherapy [178]. Currently, one oncolytic virus-based cancer therapy for metastatic melanoma is approved for clinical use by FDA (talimogene laherparepvec). The therapy uses herpesviruses modified to selectively replicate and produce granulocyte-macrophage colony-stimulating factor (GM-CSF) within tumors after direct injection, and enhance systemic antitumor immune responses [179,180]. Systemic delivery of immunogenic viruses can be disturbed by the host immune response, therefore Labani-Motlagh et al. aimed to produce immunosuppressive EVs to carry immunostimulatory molecules to induce the immune system in a predicted way. EVs derived from melanoma cells transfected with transgenes, encoded by the oncolytic viruses, were armed with costimulatory molecules CD40L and 4-1BBL [178]. 4-1BBL is endogenously expressed on activated APCs, T cells, and several carcinoma cell lines. Both ligands promote costimulatory signals leading to the activation of immature DCs and attracting T cells to the tumor [178]. Park et al. proposed immunotherapy based on co-immunization with tissue-derived EVs and synthetic bacterial vesicles of *E. coli* (SyBV), which elicited tumor regression in melanoma-bearing mice. The administration of SyBV and melanoma patient-derived EVs acted synergistically with anti-PD-L1 immunotherapy [181]. Hazekawa et al. showed that autologous serum-derived exosomes loaded with anti-Glypican-3-(GPC3) siRNA significantly decreased the number of metastatic lung cancer colonies [182]. The suppression of metastasis was also obtained by using EVs derived from metastatic melanoma cells that overexpressed Nanog [183]. Hu et al. used exosomes loaded with superparamagnetic iron oxide nanoparticles (SPION) for magnetic resonance tracking to predict which lymph nodes could be colonized by melanoma cells in a mouse model [184]. Zhuang et al. fused SPION-loaded exosomes with cell-penetrating peptides (CPP) and TNF-α (CTNF-α-Exo-SPION). This combination increased the binding capacity of TNF-α to receptor TNFR I and induced TNF-α mediated apoptotic pathway in a murine tumor model [185]. In turn, Hu et al. produced fibroblast-activation-protein gene-engineered tumor-derived exosome-like nanovesicles (eNVs-FAP) by the extrusion of fibroblast activation protein-α (FAP)-overexpressing melanoma cells. eNVs-FAP were used for mouse immunization before or after the inoculation of cancer cells. The result showed that eNVs-FAP induced protective antitumor immunity by induction of ferroptosis that resulted in increasing survival time, decreasing tumor volume, and decreasing the number of FAP+ CAFs in tumor environment [186]. Lee et al. showed that by using simultaneously exosomes packed with SD-208, an inhibitor of the transforming growth factor-β receptor I (TGFβRI) and resiquimod (R848), an agonist of the toll-like receptor (TLR)-7/8 were capable to suppress melanoma growth in vivo [187].

### 8.2. EVs as a Part of Treatment Modalities against Melanoma

Several clinical trials to verify the potential use of autologous exosomes derived from DC pulsed with tumor peptides or RNA for the immunization of cancer patients as cell-free vaccines have been designed [188]. Most recently, a clinical trial (NCT04581382) with therapeutic plasma exchange in metastatic melanoma has been launched to restore antimelanoma immunity and improve outcomes of anti-PD-L1 immunotherapy [189].

## 9. Conclusions

Exosomes and other EVs are involved in cancer progression, support the immune evasion, and the development and spreading of drug resistance. As present in biofluids, they can be used as non-invasive biomarkers of cancer development and treatment efficacy, whereas their properties, such as prolonged half-life in the circulation and low immunogenicity, predispose them to serve as vehicles for therapeutic agents. High-throughput databases providing information on a molecular cargo of different types of EVs, including EVpedia [190], Exocarta [191,192], and Vesiclepedia [193,194], have been created and expanded. While more needs to be done to overcome the challenges related to the purification and heterogeneity of obtained EVs, as well as their efficient application as diagnostic tools and vehicles for therapeutic agents, the growth of the EV field in terms of research and development is astonishing [195].

## Figures and Tables

**Table 1:** The role of EVs in melanoma resistance to targeted therapy and immunotherapy with checkpoint inhibitors.

EV Source	Functional Molecule(s) in EVs	Main Finding	Type of Study	Ref.
resistance to targeted therapy				
culture of vemurafenib-treated cells	miR-211-5p (elevated)	increased proliferation of PLX-resistant cells	in vitro in vivo	[102]
plasma of patients pre- and on-treatment with BRAFi and MEKi	let-7g-5p miR-497-5p (elevated)	higher probability of patient response to BRAFi and MEKi	clinical observation	[103]
culture of vemurafenib-treated cells	miR-3613-3p (reduced)	enhanced activation of CDC7 and ERK1/2 in resistant cells	in vitro	[104]
plasma of patients resistant to BRAFi and MEKi	BRAF splicing variant	associated with resistance	clinical observation	[105]
culture of vemurafenib-treated cells	PDGFRβ (elevated)	increased viability of sensitive cells in the presence of the drug	in vitro	[106]
culture of vemurafenib-treated cells	ALK^RES^	spreading drug resistant phenotype to sensitive cells	in vitro	[107]
resistance to immunotherapy				
plasma of patients treated with nivolumab and pembrolizumab	PD-L1 mRNA (decreased)	patients responding to therapy	clinical observation	[108]
plasma of patients (prospective study; EXOMEL cohort)	PD-L1 protein (elevated)	progression on therapy	clinical observation	[109]
plasma of patients (prospective study)	PD-L1, PD-1 proteins	nivolumab binds to PD-1-positive EVs	clinical observation	[110]
culture of xCT inhibitor-treated cells; blood samples of mice; plasma of patients	xCT (elevated)	inhibition of xCT suppressed proliferation and progression, and diminished therapeutic effect of the anti-PD-L1 mAb	in vitro in vivo clinical observation	[111]
serum of patients treated with nivolumab or pembrolizumab	CD73	significantly increased in non-responders	clinical observation	[75]
blood samples from patients prior to treatment	uPAR (elevated)	innate resistance to checkpoint inhibitors	clinical observation	[112]

ALK^RES^, a truncated form of anaplastic lymphoma kinase; BRAFi, inhibitor of BRAF^V600^; CDC7, cell division cycle 7; MEKi, inhibitor of MEK1/2; PDGFRβ, platelet-derived growth factor receptor beta; PD-L1, programmed cell death ligand 1; PLX, vemurafenib; uPAR, urokinase-type plasminogen activator receptor; xCT, cystine/glutamate antiporter.
